# Engineering Baker’s Yeast for Efficient cAMP Synthesis via Regulation of PKA Activity

**DOI:** 10.3390/foods14091533

**Published:** 2025-04-27

**Authors:** Xiaomeng Fu, Kunqiang Hong

**Affiliations:** 1 College of Food Science and Technology, Wuhan Business University, Wuhan 430056, China; 1019207099@tju.edu.cn; 2 College of Food Science and Engineering, Wuhan Polytechnic University, Wuhan 430023, China

**Keywords:** cAMP, nutritional yeast, promoter truncation, *TPK*, *BCY1*, PKA activity

## Abstract

cAMP (cyclic adenosine-3′,5′-monophosphate) has extensive physiological functions and nutritional value for living organisms, and it regulates cellular metabolism mainly by modulating PKA (protein kinase A) activity. The current yields of cAMP synthesized by microbial fermentation are still low, which is arousing interest in developing high-yield cAMP strains. In this work, two baker’s yeasts with high cAMP content were constructed by knocking out *BCY1*, *TPK3*, and *TPK2 genes*, and truncating the promoter of the *TPK1 gene*. The content of cAMP in BN5-126 and BN5-310 (with the *TPK1* gene promoter truncated by 126 and 310 bp in BN5) was improved by 30- and 9-fold, respectively, relative to the wild strain. The *TPK1* gene mRNA levels of BN5-126 and BN5-310 were decreased by 18% and 40%, respectively, without significant changes in growth performance. The results of heat shock tolerance of engineered strains also reflected the enhanced PKA activity. This work demonstrates a novel strategy for regulating gene expression to boost cAMP biosynthesis in yeast, providing a promising platform for producing nutritionally enriched and functional fermented products.

## 1. Introduction

cAMP (cyclic adenosine-3′,5′-monophosphate) is a nucleotide derivative found widely in living organisms. It acts as a secondary messenger for a variety of hormones to regulate a series of biochemical reaction processes within the cells of organisms. It is found in trace amounts in plants, animals, and microorganisms and can regulate cellular physiological activities and material metabolism. It is also the central effector of the cAMP signaling pathway [1]. cAMP has also shown important medicinal value because of its strongly functional properties, which have prompted the research in cAMP synthesis. It plays an important role in anti-cancer, anti-virus, myocardial ischemia, and anemia; it can be applied for regulating the blood sugar level and fat metabolism, accelerating body fat decomposition, reducing fat accumulation, and preventing food allergies [2,3].

cAMP is rich in jujube. However, the low yield of cAMP extracted from jujube could not meet the needs of industrial production. Currently, most of the cAMP sold in the market are chemically synthesized products, and the chemical synthesis process is complicated and exists toxic by-products [4]. With the enhancement of our living standards and the improvement of environmental protection awareness, a more environmentally friendly cAMP synthesis method has a broad market demand in the functional nutrition foods. Microbial fermentation for the production of natural products is a widespread topic in recent years, and its social benefits and environmental protection benefits are very obvious.

Currently, *Myxococcus xanthus*, *Bacillus subtilis*, *Escherichia coli*, and *Saccharomyces cerevisiae* have been reported to be used for cAMP synthesis studies. The cAMP content in *B. subtilis* was enhanced significantly by modulating the DO (dissolved oxygen) during fermentation. When DO was controlled at 0–10%, cAMP reached 19.2 g L^−1^ at 52 h of fermentation [5]. The intracellular cAMP concentration in *M. xanthus* increased quickly under starvation and inducement of glycerol [6]. Notleymcrobb, L., et al. found that *E. coli* fermented in a minimal medium that includes micromolar glucose could lead to a 10-fold higher intracellular cAMP concentration than in a medium with excess glucose [7]. Although the above studies have provided further insights into the synthesis of cAMP, the yield of cAMP remains low in prokaryotic microorganisms.

**Figure 1:**
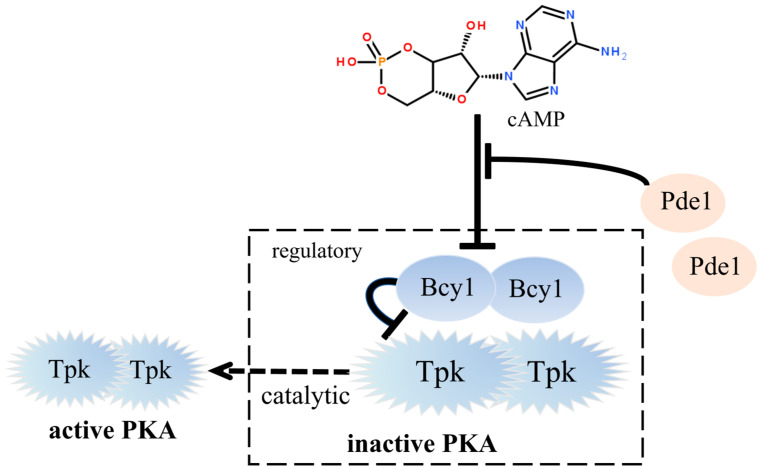
cAMP signal pathway in baker’s yeast.

As a eukaryotic organism, *S. cerevisiae* has a clear genetic background and mature strain modification techniques. The baker’s yeast was used commonly in the modern food industry for the production of bread, steamed buns, and crackers. The construction of functional baker’s yeast has important significance for the development of functional foods. For example, high chromium functional yeast could decrease the epididymal adipose levels in the obese model [8], and the functional baker’s yeast could improve the nutritional protein value of home-baked bread used by low-income communities [9]. Therefore, construction of high cAMP yield functional yeast was feasible and realizable. Research on the synthesis, breakdown, and feedback inhibition mechanisms of intracellular cAMP in Saccharomyces cerevisiae has yielded significant insights [10]. Investigations revealed that merely a 10% readthrough at the PDE2 stop codons could effectively prompt a substantial elevation in cAMP concentration in *S. cerevisiae* [11]. cAMP content could be enhanced by glucose-induced activation and application of substances inducing cytoplasmic acidification, such as 2,4-dinitrophenol in *S. cerevisiae* [12]. As shown in Figure 1, the intracellular Ras-cAMP signaling pathway is tightly negatively feedback regulated by PKA. Reports showed that cells with high PKA activity failed to accumulate glycogen, sporulate, and heat shock resistance [13]. Under normal conditions, the zinc finger transcription factors Msn2/4 and the protein kinase Rim15 are involved in regulating the cellular stress response. When PKA activity is too high, its negative regulation of Msn2/4 and Rim15 is enhanced, which leads to a decreased ability of the cells to respond to heat stress and ultimately reduces their heat stress tolerance. The intracellular cAMP levels could be enhanced by modulating PKA (protein kinase A) activity [14]. PKA regulates intracellular cAMP levels at both synthetic and catabolic levels by phosphorylating different proteins in the Ras-cAMP pathway and leading to changes in the activity of these proteins. The PKA complex is composed of a regulatory subunit (originating from the BCY1 gene) and catalytic subunits (stemming from the TPK1, TPK2, and TPK3 genes), which exhibit feedback inhibition on the accumulation of cAMP. TPK genes have similar and overlapping functions; the mutation inactivation or destruction of any two of the TPK genes would not affect the cell phenotype, but the deletion of three genes at the same time was lethal for cells [15]. The modulation of the *TPK1*, *TPK2*, and *TPK3* gene expression level can regulate the PKA activity and enhance the cAMP levels in *S. cerevisiae* [16,17]. The regulatory subunit was the predominant cAMP-binding protein; its deletion resulted in the deficiency of cAMP-dependent protein kinase and accumulated high levels of cAMP. Therefore, in order to achieve a substantial accumulation of cAMP in *S. cerevisiae* while not affecting the normal growth of yeast cells, it is necessary to employ metabolic engineering strategies to finely regulate the expression activity of the *BCY1* and *TPK* genes.

In this study, strains with enhanced heat shock tolerance and remarkably increased cAMP levels were obtained by the abolishment of the feedback regulation in PKA. Increased cAMP levels optimized the metabolic pathways of yeast, enabling it to exhibit higher stability and adaptability in complex industrial environments, thereby enhancing fermentation efficiency. This study provides new insights for the development of novel industrial yeast strains and is of significant reference value for the production of high-value-added biological products and construction of functional yeast.

## 2. Materials and Methods

### 2.1. Strains, Plasmids, and Medium

Strains and plasmids used in this study are listed in Table 1. The BYN strain, derived from the sporulation of industrial diploid baker’s yeast, serves as the chassis strain. It is cultured at 30 °C in YPD medium (comprising 10 g L^−1^ yeast extract, 20 g L^−1^ peptone, and 20 g L^−1^ glucose) or SC-ura3 medium (containing 6.7 g L^−1^ yeast nitrogen base without amino acids, supplemented with all required auxotrophic factors except uracil, and 20 g L^−1^ glucose). The W303-1a strain (*MATa ade2 ura3 leu2 trp1 his3 can1*) was employed to amplify the mutant *ura3* gene, which was then introduced into baker’s yeast BN, yielding the ura3 mutant strain BN1. 5-Fluoroorotic acid (5-FOA) medium (composed of 6.7 g L^−1^ yeast nitrogen base without amino acids, supplemented with all necessary auxotrophic requirements, 20 g L^−1^ glucose, and 2 g L^−1^ 5-fluoroorotic acid) was utilized exclusively for the selection of uracil auxotrophic transformants. Yeast chromosomal DNA was isolated by a yeast genomic DNA extraction kit (Beijing Solarbio Science and Technolog, Beijing, China). *DH5ɑ* was used for plasmid construction, which was cultivated at 37 °C in LB medium (10 g L^−1^ tryptone, 5 g L^−1^ yeast extract, and 5 g L^−1^ NaCl, pH 7.0) with added ampicillin (100 mg L^−1^) for plasmid screening. PCR primers utilized in this investigation were devised based on the genomic sequence of *S. cerevisiae* S288c (obtained from NCBI, http://www.ncbi.nlm.nih.gov/) and are detailed in Table S1. All oligonucleotide primers were manufactured and purified by Genewiz Biotechnology Corporation (Suzhou, China).

### 2.2. Construction of Engineered Strains

BN1 is a uracil-deficient strain obtained by replacing the normal *URA3* gene in BN with the mutational *ura3* gene through lithium acetate transformation [18]. Transformants were cultivated in SC-5FOA solid plates to screen strains with mutational *ura3*, resulting in strain BN1. Deletion of *TPK3*, *BCY1*, and *TPK2* genes were operated in the same way. The *TPK3* gene was replaced by the *URA3* gene in BN1 and screened on the SC-URA3 solid media, resulting in BN2. The *BCY1* gene was replaced by the *KanMX* gene amplified by PCR from *pUG6* in BN2 and screened on YPD solid plates containing 0.33% (V/V) G418 [19], resulting in BN3. The *TPK2* gene was replaced by the *HPH* gene (the *HPH* gene was amplified from *PDH25*) in BN3 [20], and transformants were plated on YPD solid medium supplemented with 0.255% (100 mg mL^−1^) hygromycin B, resulting in BN4. The *URA3* gene of BN4 was replaced by a mutational *ura3* sequence, which resulted in engineered strain BN5.

**Figure 2:**
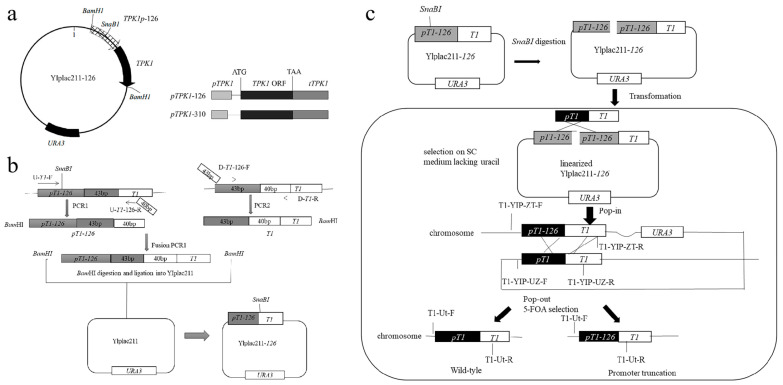
Protocol for the dual-stage integration technique utilized in promoter truncation. (**a**) Schematic representation of the *TPK1* gene promoter engineering strategy. (**b**) Construction of the vector YIplac211-126. YIplac211-310 was operated in the same way. The fragments pT1-126 and T1 in the BY9a genome were amplified through PCR1 and PCR2, respectively. Fusion PCR was performed by mixing the PCR1 and PCR2 products as templates in PCR3 to obtain pT1-126-T1. After digestion by *KpnI*, the fusion fragment pT1-126-T1 was introduced into YIplac211, yielding YIplac211-126. The upstream region of pT1-126 was employed for restriction enzyme digestion before transformation. (**c**) Schematic depiction of the dual-stage integration technique. YIplac211-126 was digested with SpeI and incorporated into the chromosome of BN5. Subsequently, the transformants grown on SC medium lacking uracil were spread on 5-FOA plates again to obtain BN5-126. The strain BN5-310 was operated in the same way.

Weakening of the *TPK1* gene expression level was achieved by two-step homologation reorganization (Figure 2) [21]. Recombinant plasmids with 3′-end truncated in the *TPK1* promoter were constructed first. As shown in Figure 2, YIplac211 was used as a vector, and primer pairs U-T1-F/U-T1-126-R and U-T1-F/U-T1-310-R were used, respectively, to amplify fragments pT1-126 and pT1-310 of BY9a. Primer pairs (D-T1-126-F/D-T1-R and D-T1-310-F/D-T1-R) were utilized to amplify the T1 fragments (-126 and -310) from the BN genome. Subsequently, the overlapping sequences functioned as primers for extending the subsequent chimeric genes. Ultimately, the fusion PCR products, purified via gel extraction, were subjected to *KpnI* digestion and ligated into the *KpnI*-digested plasmid YIplac211, thereby generating the recombinant plasmids YIplac211-126 and YIplac211-310 (Figure 2b). YIplac211-126 and YIplac211-310 were linearized with *Spe*I and transformed into BN5. Transformants were cultivated in uracil-free SC medium and incubated in YPD liquid medium at 30 °C for one day; then, 100 μL of suspension (100-fold dilution) was spread on the SC medium. The mutant ura3 was restored to wild type through transformation with the wild-type URA3 sequence, after validation of the transformants by colony PCR. Finally, the engineered strains BN5-126 and BN5-310 were obtained (Figure 2c).

### 2.3. Measurements of Intracellular cAMP in Engineered Strains

The detection method of cAMP in *S. cerevisiae* was referenced from the report by Bai Xiaojia et al. with minor modifications [22]. Preparation of solution: 50 mmol L^−1^ Tris-HCl solution: A 100 mL volumetric flask was filled with 5 mL of 1 mol L^−1^ Tris-HCl, followed by dilution with ultrapure water and adjustment of the pH to 7.4; 31.25 mmol L^−1^ Na_2_ATP solution: Dissolving 1.89 g Na_2_ATP in ultrapure water, make up to 100 mL, frozen storage; 31.25 mmol L^−1^ MnCl_2_ Solution: 0.62 g of MnCl_2_ were dissolved in ultrapure water to a volume of 100 mL; 0.31 mol L^−1^ MES buffer: 6.10 g MES were dissolved in ultrapure water, adjusting pH to 7.0 and setting volume to 100 mL; 1 mg mL^−1^ cAMP standard solution: 0.1 g cAMP standards were dissolved with 50 mmol L^−1^ potassium dihydrogen phosphate solution, setting volume to 100 mL; 50 mmol L^−1^ potassium dihydrogen phosphate solution: 6.80 g potassium dihydrogen phosphate were dissolved in ultrapure water and diluted to 1 L.

Pretreatment of yeast cells: Yeast cells were cultured in 100 mL YPD liquid medium at 180 rpm, 30 °C for 12 h. For determination of dry weight of cells, 10 mL of broth was transferred into a 10 mL centrifuge tube, washed twice with sterile water, and precipitation of yeast was transferred to a weighed centrifuge tube for desiccation and recording dry weight of cells. For the extraction of cAMP, 2 mL of yeast solution were taken in an Eppendorf tube and centrifuged at 12,000× *g* rpm for 1 min. The cell precipitate was washed twice using a 50 mmol L^−1^ Tris-HCl solution, then resuspended in a mixture of 1 mL Tris-HCl solution and 200 μL dimethyl sulfoxide. The mixture was centrifuged at 12,000× *g* rpm for 1 min, after which the supernatant was discarded. The precipitate was subsequently washed twice with a 0.31 mol L^−1^ MES buffer. To the reaction mixture, 80 μL of a 31.25 mmol L^−1^ Na_2_ATP solution, a 31.25 mmol L^−1^ MnCl_2_ solution that had been preincubated at 30 °C, and 840 μL of a preheated 0.31 mol L^−1^ MES buffer were added. The mixture was incubated at 30 °C for 10 min and boiled for 1 min to terminate the reaction. After cooling, centrifugation, filtration, and determination of the cAMP content in the filtrate by liquid chromatography. The enzyme activity was quantified by the quantity of product (μg mL^−1^) generated from the reaction of 1 mg of dry cells with Na_2_ATP at 30 °C for 1 min.

Detection of cAMP: HPLC (High Performance Liquid Chromatography) was used to detect cAMP. Chromatographic conditions: ZORBAX Eclipse Plus C18 column (250 mm × 4.6 mm, 5 μm); Acetonitrile: Potassium dihydrogen phosphate solution (50 mmol L^−1^) = 20:80; Wavelength: 254 nm; Flow rate: 0.8 mL min^−1^; Column temperature: 25 °C; Injection volume: 20 μL; Quantitative method: Quantitation by peak area using the external standard method. A quaternary pump system was used for avoiding cold-precipitated buffer.

### 2.4. RT-qPCR Assay

Detection of transcriptional activity in the logarithmic growth period of BN, BN2, BN3, BN4, BN5-126, and BN5-310 was operated as described previously [23], using primer pairs TPK3-F/TPK3-R, BCY1-F/BCY1, TPK1-F/TPK1-R, TPK2-F/TPK2-R, and ACT1-F/ACT1-R, respectively (Table S1).

### 2.5. Determination of Heat Shock Resistance in Engineered Strains

The heat shock resistance was assessed to indicate the PKA activity of engineered strains, and the method was referenced from the report by Bai Xiaojia et al. with minor modifications [22]. Initially, the strains were cultivated in YPD broth at 30 °C until they reached the exponential growth phase, at which point the OD_600_ of the cells was standardized to 1.0 with sterile YPD broth. Subsequently, the cell suspensions were subjected to heat shock for 3 min in a 56 °C water bath. Aliquots of 1 µL from various dilutions of the cell suspensions were then plated onto YPD agar plates. These plates were incubated at 30 °C for 2–3 days, after which they were photographed, and the growth morphology was evaluated. Cell suspensions before and after treatment were spread on YPD solid plates, and the survival rate of engineered strains under heat shock conditions was assessed by counting the number of colonies.

## 3. Results

### 3.1. Construction of Engineered Strains

In *S. cerevisiae*, the intracellular cAMP accumulation was regulated negatively by PKA activity. In order to enhance the cAMP level of cells, we attempted to modulate the expression levels of relevant genes encoding regulatory and catalytic subunits of PKA. The *BCY1* gene encodes the regulatory subunit of PKA, while the TPK genes (*TPK1*, *TPK2*, and *TPK3*) encode the catalytic subunits of PKA. Deletion of *BCY1* and two of the TPK genes had no effect on strain activity, but simultaneous deletion of TPK genes was lethal for strains [24]. Therefore, the PKA activity was intended to decrease for cAMP accumulation by deleting *BCY1*, *TPK3*, and *TPK2* genes and attenuating the expression level of the *TPK1* gene. The *TPK3* gene was deleted in BN1 using homologous recombination, obtaining BN2. The *BCY1* gene was deleted in BN2, and BN3 was obtained. The *TPK2* gene was deleted in BN3, and BN4 was obtained. The weakening of the *TPK1* gene was realized by truncation of the *TPK1* promoter from the 3′-end by 126 bp and 310 bp, respectively. The weakened strains BN5-126 and BN5-310 were obtained by transferring the linearized recombinant plasmid to strain BN5, using the method of two-step homologous integration.

### 3.2. cAMP Levels and Growth Performance of Engineered Strains

**Figure 3:**
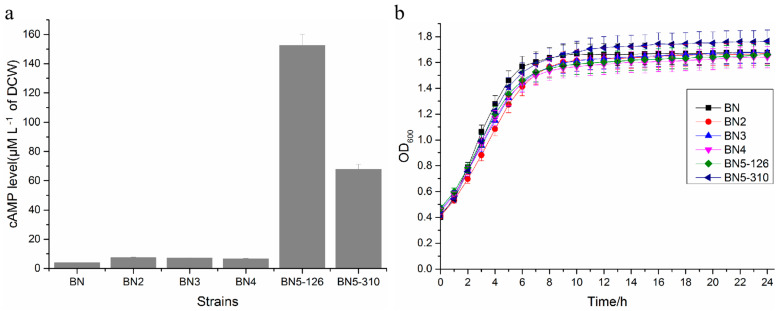
cAMP concentrations within engineered strains. (**a**) cAMP levels of engineered strains. (**b**) Growth characteristics of engineered strains in YPD broth incubated at 30 °C for 24 h. Data are depicted as the average values and standard deviations from three separate biological experiments. Error bars indicate ± SD.

cAMP levels of engineered strains were detected by HPLC. As shown in Figure 3a, the cAMP levels of BN2, BN3, and BN4 have slightly elevated compared to BN. It shows that the deletion of *TPK3*, *BCY1*, and *TPK2* genes have little effect on the intracellular cAMP levels. Strains with weakened *TPK1* genes had an obvious elevation, and the intracellular cAMP levels of BN5-126 and BN5-310 were increased about 30- and 9-fold, respectively, compared to wild-type strains (Figure 3a), which illustrated that the intracellular cAMP levels were associated with the weakened degree of the *TPK2* gene. The cAMP yield of BN5-126 (152.53 µM L^−1^ of DCW) also reached the highest level in yeast. Research showed that the growth performance of strains was correlated with PKA activity [9]. To verify the effect of the deletion and weakening of genes encoding PKA activity on strain growth, growth performance of strains was operated in YPD liquid medium at 30 °C for 24 h by a fully automatic growth curve meter in the absorbance value of OD_600_. From the growth curves of engineered and parent strains in Figure 3, no significant growth differences are observed between the two, which suggests that the deletion of *BCY1* and *TPK2/3* and the weakening of *TPK1* did not significantly influence the growth performance of strains. These data also showed that the comprehensive regulation of PKA activity can increase the intracellular cAMP levels significantly without influencing growth performance.

### 3.3. Heat Shock Resistance of the Engineered Strains

**Figure 4:**
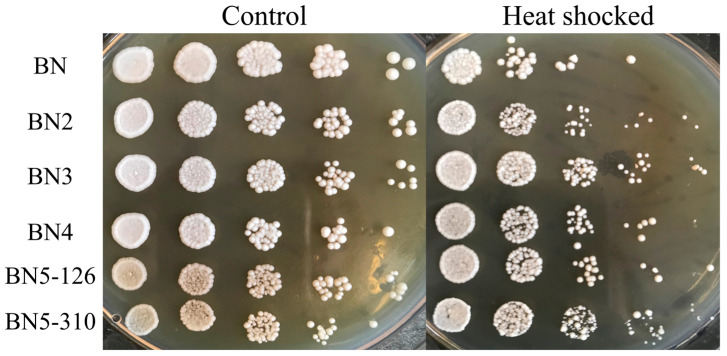
The heat shock resistance of engineered strains. Cells were either heat shocked at 56 °C for 3 min or left untreated, then subjected to a 10-fold serial dilution and spotted onto YPD plates. The plates were incubated at 30 °C for 3 days and subsequently photographed.

In general, strains with downgraded PKA activity could exhibit increased heat shock resistance [13]. So, the heat shock resistance of engineered strains was detected at 56 °C for 3 min to reflect the PKA activity. As shown in Figure 4, the growth performance of the strains was significantly affected after treatment; the colonies following heat shock treatment are smaller in size compared to those of the control strain. From left to right, as the dilution factor increases, the number of colonies in both groups decreases. However, after heat shock treatment, the number of colonies of BN5-310 is greater than that of the control at any dilution factor, which indicates that BN5-310 exhibited stronger heat shock resistance relative to others. The survival rate experimental results in Table S2 also indicate the high heat shock tolerance of BN5-310. The results above demonstrated that combined regulation of *BCY1* and *TPK* genes could enhance heat shock resistance of engineered strains, which also reflected the reduced PKA activity.

### 3.4. Relative Expression Level of TPK1 Gene in Engineered Strains

**Figure 5:**
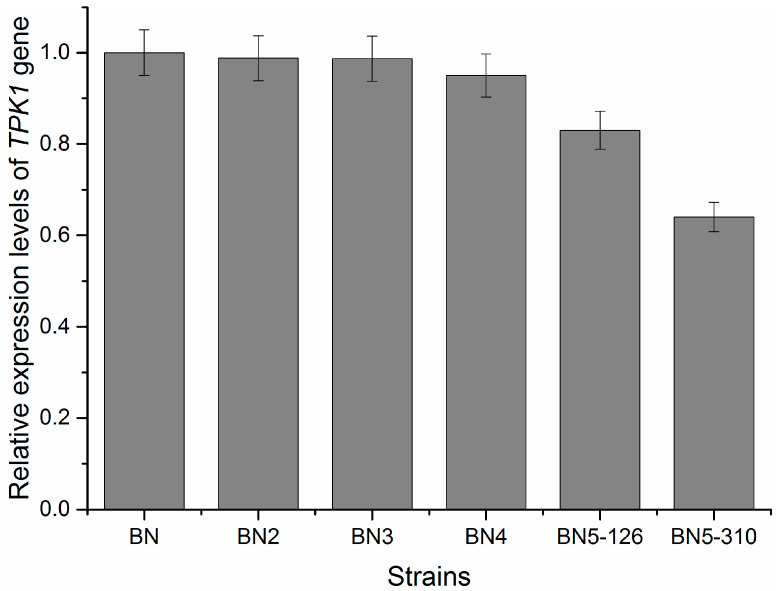
Relative expression levels of the *TPK1* gene in engineered strains. The mRNA levels of the *TPK1* gene were assessed via RT-qPCR, with the experiments conducted in triplicate. The data presented are the mean values from three independent experiments, with error bars indicating ± SD.

Relative expression levels of the *TPK1* gene were detected using the *ACT1* gene as a positive control. Relative expression levels of the *TPK1* gene in engineered strains were assessed to determine the influence of *TPK1* gene promoter truncation on *TPK1* transcription levels. In Figure 5, the expression levels of the *TPK1* gene in BN5-126 and BN5-310 approximately decreased by approximately 18% and 40% compared to BN, respectively. The illustration indicates a positive correlation between promoter length and the expression level of the *TPK1* gene.

## 4. Discussion

As a kind of edible yeast, industrial baker’s yeast is widely used in food production. To be more acceptable to customers, it is necessary to ensure the safety of strains. The method of two-step integration is an effective and safe method in gene editing and leaves engineered strains without residue of exogenous genes [25]. Genetic engineering for fine-tuning enzyme activity has been documented as an effective approach to enhance target product yields [26,27]. Moreover, our prior research has shown that truncating the promoter of a target gene can effectively modulate the associated enzyme activities [28]. The decreased PKA activity is often accompanied by the decrease of growth performance [29]; therefore, the fine-tuning could increase the target products to a large extent and not affect the growth and fermentation of strains.

Although there were many researches on increasing the intracellular cAMP level in different host strains, including *M. xanthus* [6], *E. coli* [30,31], and *B. subtilis* [32], the baker’s yeast used in our work is an industrial strain that differs from the previously reported laboratory strain [17]. Generally speaking, laboratory strains are well-documented and easy to manipulate. However, laboratory strains are physiologically different from industrial strains [33]. Industrial strains typically possess high tolerance and efficient growth and fermentation performance, which gives them an advantage in the large-scale production of natural products [34]. The differences between industrial strains and laboratory strains usually blocks the transformation from theoretical research to practical application. Thus, the outcomes derived from engineering industrial strains are evidently more aligned with industrial applications. Further enhancement of the intracellular cAMP level can be achieved through optimization of the biosynthetic pathway and the genetic networks of the host strain, as well as optimization of the fermentation process.

As a raw material for cAMP, jujube is usually faced with problems of long growth cycle, complicated extraction process, and low efficiency [35], and yeast with high cAMP yield is relatively more economical, more effective, and more productive [36]. Yeasts could be used to produce a variety of nutrients as a nutritious food. Ding et al. obtain a nutritious edible yeast named M3-20 through three-step continual EMS mutation, the riboflavin content of which saw a 215% increase relative to the parent strain [37]. Branduardi et al. reported that the production of L-ascorbic acid from D-glucose by metabolically engineered yeast cells [38]. Similarly, yeast can also be used to produce cAMP as a nutritional yeast with a high yield of cAMP. The cAMP levels of the engineered strains in this study were increased up to 30-fold relative to the wild-type, which is the highest yield of cAMP in yeast. Nutritional yeast of high cAMP yield has been mainly studied theoretically [15,17], and this marks the first instance of synthesizing cAMP in industrial baker’s yeast through genetic engineering. This breakthrough of the work had a great value in the enrichment of nutritional yeast.

In conclusion, this is the first time we have constructed industrial and nutritional baker’s yeast with high cAMP yield. Our results showed that increasing intracellular cAMP accumulation in yeast by modulating PKA activity is a very effective approach in baker’s yeast. The findings from our research using industrial baker’s yeast may better reflect industrial application scenarios and could enhance the transition from lab discoveries to industrial-scale production. The increase in cAMP levels in engineered strains also enhances their tolerance, enabling them to better adapt to various stress conditions commonly encountered in industrial fermentation processes, such as high temperatures and high osmotic pressure. Compared to traditional cAMP synthesis methods, this approach has the advantages of simple operation, low raw material costs, and environmental safety, making it more suitable for industrial production. However, genetically modified yeast may experience unstable gene expression and blocked metabolic pathways during long-term fermentation, which can affect its performance. Therefore, improving the stability and robustness of cAMP-producing strains in large-scale fermentation is an important part of future cAMP synthesis research.

## Figures and Tables

**Table 1:** Strains and plasmids used in this study.

Strain	Genotype	Source
BYN	Industrial baker’s yeast	Our lab
BN	baker’s yeast, MATa	This study
BN1	MATa, ura3 was inserted in the URA3 site	This study
BN2	MATa, TPK3 was deleted and URA3 was inserted	This study
BN3	BN2, BCY1 was deleted and KanMX was inserted	This study
BN4	BN3, TPK2 was deleted and HPH was inserted	This study
BN5	BN4, ura3 was inserted in the URA3 site	This study
BN5-126	BN5, URA3 and TPK1p−126 were inserted in the ura3 site	This study
BN5-310	BN5, URA3 and TPK1p−310 were inserted in the ura3 site	This study
Plasmid	Genotype	Source
YIplac211	Ampr, URA3	Our lab
YIplac211-126	YIplac211, pTPK1−126-TPK1-tTPK1	This study
YIplac211-310	YIplac211, pTPK1−310-TPK1-tTPK1	This study
DH5ɑ	supE44 DlacU169(u 80lacZDM15) hsdR17 recAl endAl gyrA96 thi-1 relA	Beyotime Biotech Ltd.
pUG6	Ampr, KanMX	Our lab
pDH25	Ampr, HPH	Our lab

## Data Availability

The original contributions presented in this study are included in the article/Supplementary Materials. Further inquiries can be directed to the corresponding author.

## Supplementary Materials

The following supporting information can be downloaded at: https://www.mdpi.com/article/10.3390/foods14091533/s1, Table S1. Primers used in this study. Table S2. Survival rate to heat shock of engineered strains. Table S3. Abbreviations and full names in manuscript.
