# Supplementary material for: Engineering Baker’s Yeast for Efficient cAMP Synthesis via Regulation of PKA Activity
Source: Foods. 2025 Apr 27;14(9):1533. doi: 10.3390/foods14091533 (PMC12071475; doi:10.3390/foods14091533)
Supplement: Supplementary file 1 [file foods-14-01533-s001.zip › foods-3560165-supplementary.pdf]

Table S1. Primers used in this study.

Primers	Sequence (5'-3')
TPK3-U-F	CGTTCCAGGTACGAGTGATT
TPK3-U-R	TTATATGTAGCTTTCGACATTTTGTGCAGGCTCGCTCTTTCCTTGT
URA3-F	AGGAAAGAGCGAGCCTGCACAAAATGTCGAAAGCTACATATAAGG
URA3-R	AATTACAATTATCCCACTGAACCTCCTTAGTTTTGCTGGCCGCAT
TPK3-D-F	TGAGAAGATGCGGCCAGCAAAACTAAGGAGGTTCAGTGGGATAAT
TPK3-D-R	CGTTCTTTATCCTTATCACG
BCY1-U-F	GAACCAAATGTCATTTCTAACG
BCY1-U-R	CTGCAGCGTACGAAGCTTCAGCTGCGTTTATTCTTACTGTTGTCT
kan-F	TCAAAAGACAACAGTAAGAATAAACGCAGCTGAAGCTTCGTACGC
kan-R	TTAAGATCGCTTCCCCTTTTTACGCATAGGCCACTAGTGGATCTG
BCY1-D-F	GATATCAGATCCACTAGTGGCCTATGCGTAAAAAGGGGAAGCGATC
BCY1-D-R	GAGGTACACGCTGGAGATACCG
Tpk2-U-F	CTGAACCGTTACTACAGCAGC
Tpk2-U-R	GCGGTGAGTTCAGGCTTTTTCATACCGACAATTTTCAACAGTATG
T2-PHP-F	CACATACTGTTGAAAATTGTCGGTATGAAAAAGCCTGAACTCACCG
T2-PHP-R	TTGTGTTTTTTGGTTCATGGAACCTATTCCTTTGCCCTCGGACG
Tpk2-D-F	CACTCGTCCGAGGGCAAAGGAATAGGTTCCATGAACCAAAAAACA C
Tpk2-D-R	GTTTTCAACTCATTAAAACGG
U-T1-F	CTAGAGGATCCCCGGGTACCGGGCTATTGTTCTTACTGC

U-T1-126-R	GACTTTTGACCACCTCCATTTTGTTCTTCAGTCGACATGTATAGGGTCAGATTCCTTAG
U-T1-310-R	GACTTTTGACCACCTCCATTTTGTTCTTCAGTCGACATCCAGATCCCGCCTTCCCTC
D-T1-126-F	TTTAATTTGATAAAGCTAAGGAATCTGACCCTATACATGTCGACTGAAGAACAAAATGG
D-T1-310-F	CGCGCTATTAGGGGGGAGGGAAGGCGGGATCTGGATGTCGACTGAAGAACAAAATGG
D-T1-R	GTGAATTCGAGCTCGGTACCCGTATAGAATGGAGATTCCGAC
ura3-F	AGGAAAGAGCGAGCCTGCACAAAATGTCGAAAGCTACATATAAGG
ura3-R	AATTACAATTATCCCACTGAACCTCCTTAGTTTTGCTGGCCGCAT
TPK2-F	CTGGATCTTTTGGTAGGGTTCA
TPK2-R	ACGTCGTTCGTCATTGGTATGT
ACT1-F	TTATTGATAACGGTTCTGGTATG
ACT1-R	CCTTGGTGTCTTGGTCTAC
TPK3-F	CATCTCAGATAGTTCCCTTTA
TPK3-R	GGCATATAAGTATATGTGG
BCY1-F	GGAGGAGAAGTCCGTCCCCA
BCY1-R	GAAGAGTTGACCTTGTTGTCG
TPK1-F	CCAAATGGCCCATTCACAATC
TPK1-R	ACCTCATTATTTGAAATTTTCC

Table S2. Survival rate to heat shock of engineered strains.

Strains	Survival rate (%)
BN	0
BN2	1.22
BN3	10.77
BN4	12.50
BN5-126	8.26
BN5-310	11.59

Table S3. Abbreviations and full names in manuscript

Abbreviations	Full names
cAMP	cyclic adenosine-3', 5'-monophosphate
PKA	protein kinase A
DO	dissolved oxygen
5-FOA	5-Fluoroorotic acid
HPLC	High Performance Liquid Chromatography
